# Maxillofacial and dental-related injuries from a Brazilian forensic science institute: Victims and perpetrators characteristics and associated risk factors

**DOI:** 10.4317/jced.56637

**Published:** 2020-08-01

**Authors:** Carlos-Diego Lopes Sá, Paulo-Goberlânio-de Barros Silva, Adriana-de Moraes Correia, Eduardo-Costa-Studart Soares, Tácio-Pinheiro Bezerra, Radamés-Bezerra Melo, Heide-dos Santos Bitú, Fábio-Wildson-Gurgel Costa

**Affiliations:** 1 DDS, MSc, PhD. Adjunct Professor, Division of Oral and Maxillofacial Surgery, School of Dentistry, Paulo Picanço School of Dentistry, Fortaleza, Brazil; 2 DDS, MSc, PhD. Adjunct Professor, Division of Oral Pathology, School of Dentistry, University Center UNICHRISTUS, Fortaleza, Brazil; 3 DDS, MSc. Postgraduate student, Division of Dental Forensic Science, School of Dentistry, University Center UNICHRISTUS, Fortaleza, Brazil. Forensic Odontologist, Perícia Forense do Estado do Ceará, Fortaleza, Brazil. Professor, Division of Dental Forensic Science, School of Dentistry, University Center UNICHRISTUS, Fortaleza, Brazil; 4 DDS, MSc, PhD. Full Professor, Division of Oral and Maxillofacial Surgery, Walter Cantídio University Hospital, Federal University of Ceará, Fortaleza, Brazil; 5 DDS, MSc, PhD. Professor, Division of Oral and Maxillofacial Surgery and Division of Dental Forensic Science, University Center UNICHRISTUS, Fortaleza, Brazil; 6 DDS, Postgraduate student, Division of Oral and Maxillofacial Surgery, School of Dentistry, Paulo Picanço School of Dentistry, Fortaleza, Brazil; 7 DDS, MSc, PhD. Adjunct Professor, Division of Oral Radiology, School of Dentistry, Federal University of Ceará, Fortaleza, Brazil

## Abstract

**Background:**

Trauma due to external causes represents one of the greatest challenges for public health services in different regions around the world. This study aimed to determine the prevalence of facial trauma, associated risk factors, and classification of body injuries in individuals who underwent forensic examination in a Brazilian center.

**Material and Methods:**

Data were collected at the Ceará State Forensic Medicine unit in a 12-year period. Sociodemographic data related to the etiological agent and lesions resulting from the bodily injury were recorded.

**Results:**

Among 1,031 physical injury exams, physical aggression (*p*<0.001), male victims aged between 21 and 30 years (*p*<0.001), salaried workers (*p*<0.001), and soft tissue and dentoalveolar injuries were significant findings. Regarding aggression, domestic violence was prevalent (*p*<0.001), perpetrated by the victim’s partner (*p*<0.001), using a blunt instrument during the aggression (*p*<0.001), and directly associated with soft tissue injury (*p*<0.001). In traffic accidents, the most common type was motorcycle accident (*p*<0.001), on weekdays (*p*=0.036), at nighttime (*p*=0.134), showing a significant association with bone fractures (*p*=0.001).

**Conclusions:**

Oral and maxillofacial injuries obtained from a Brazilian forensic science center were significantly associated with sociodemographic and etiological factors.

** Key words:**Forensic dentistry, facial trauma, violence, public health.

## Introduction

Trauma due to external causes represents one of the greatest challenges for public health services in different regions around the world (1-3). Every day in developed and developing countries, thousands of people are victims of interpersonal violence or are involved in traffic accidents, overburdening health services (1-4). Trauma in the face, head and neck region is generally the most prevalent, the vast majority of which affect vulnerable groups of the population, involving an anatomical region that largely defines the perception of the individual’s self-image and identity, often associated with persistent damage (5).

Disorders of the maxillofacial complex resulting from trauma stand out from other types due to a high incidence and diversity of injuries, as well as being generally associated with a severe degree of morbidity, loss of function, and financial burden (6). In a study conducted in the United States, it was found that costs related to length of hospital stay and surgical treatment of facial fractures indicated an extensive consumption of hospital resources (7). In general terms, maxillofacial injury is a health problem that presents epidemiology, pathophysiology, morbidity and mortality investigated by several researchers (2-4). Its epidemiology can vary widely, and depends on several factors, including culture, socioeconomic status, and population density (1-8).

In this context, stressing the importance of epidemiological studies of maxillofacial trauma helps to identify the etiological profile, aids in assessing the efficiency of road safety measures, outlines the behavior pattern of people from different cultures regarding associated factors, and contributes to the establishment of preventive socio-educational measures related to public safety policies (9).

Epidemiological studies of maxillofacial injuries conducted based on records of forensic science centers are considered scarce (6). Thus, the aim of this study was to characterize maxillofacial injuries through morbidity data on victims who were taken to a forensic service unit in order to undergo medical/dental examination due to physical aggression or traffic accident.

## Material and Methods

A cross-sectional retrospective study was conducted at a center of forensic medicine and dentistry (Perícia Forense do Estado do Ceará – PEFOCE, Brazil) in a 12-year period. The present study was approved by the Research Ethics Committee of the Federal University of Ceará (Approval number: 1.893.954).

Data were collected by observing the records of expert examinations of forensic odontologists and police reports attached to expert examiner reports in order to complete a form specifically designed for the recording of data on facial trauma, recording sociodemographic data as well as data related to the etiological agent and to the injuries resulting from the trauma, and to determine the classification of the damage according to current legislation, particularly any acquired debility and/or deformity based on the experts’ answers to the questions, which is one of the items that comprise the forensic medical/dental examination.

The sociodemographic data collected were: sex, age, marital status, occupation, education level, and place of residence of the person undergoing the examination. The information related to the etiological agent was grouped into traffic accidents, physical aggression, and other.

Based on the resulting injuries, data related to facial damage were obtained, such as: soft tissue injury, specifying its location; dentoalveolar fracture, obtaining information about the type of fracture and the number of teeth affected; and bone fracture, determining the anatomical site affected according to the classification of factures of the mandible, maxilla, zygomatic-orbital complex, nasal bones, frontal complex, and naso-orbito-ethmoid (NOE) complex.

The bodily injury report is composed of questions to help obtain clarifications about the existence of an offense to the integrity of the patient’s body or health; about the possible instrument or medium that produced the offense; whether the offense was caused by any insidious or cruel means or was life-threatening; whether the offense resulted in incapacity for one’s usual occupations for more than thirty days; permanent debility, loss or disuse of a limb, sense or function; or permanent incapacity for work, incurable illness, or permanent deformity. In the expert forensic examination, the answers should be clear and are usually stated as “YES/NO” when there is a positive or negative conviction regarding the respective question; “NO ELEMENTS” when the respondent does not have the conviction to answer either yes or no to the question; “IMPAIRED” when there is any impossibility in answering the question; or “AWAIT” when the answer depends on further examination or subsequent reevaluation.

The data were expressed as absolute and percentage frequency or mean and standard deviation, and compared using Fisher’s exact test and Pearson’s chi-square test (categorical data), or Mann-Whitney and Kruskal-Wallis/Dunn tests (non-parametric data), respectively. A multinomial logistic regression model was used with the variables that showed significant association in the bivariate analysis. All analyses were carried out adopting 95% confidence using Statistical Package for Social Sciences (SPSS) software (version 20.0 for Windows®).

## Results

-Characterization of the sample

In all, 1,031 bodily injury examinations were documented in the period from 2006 to 2017. Of these, the most prevalent were physical injury examinations, with 461 (44.7%) cases, followed by other injuries (n = 215, 20.9%), traffic accidents (n = 208, 20.2%), and supplementary examinations for reevaluation of bodily injury (n = 147, 14.3%). The most prevalent age group of the victims was 21-30 years (n = 109, 33.2%), males (n = 595, 58.4%), and marital status ‘single’ (n = 192, 67.4%). The most affected were salaried workers (n = 121, 54.5%), with high school diploma (n = 58, 33%) and etiological factor resulting from physical aggression (n = 501, 64.3%) (Table 1).

**Figure 1:**
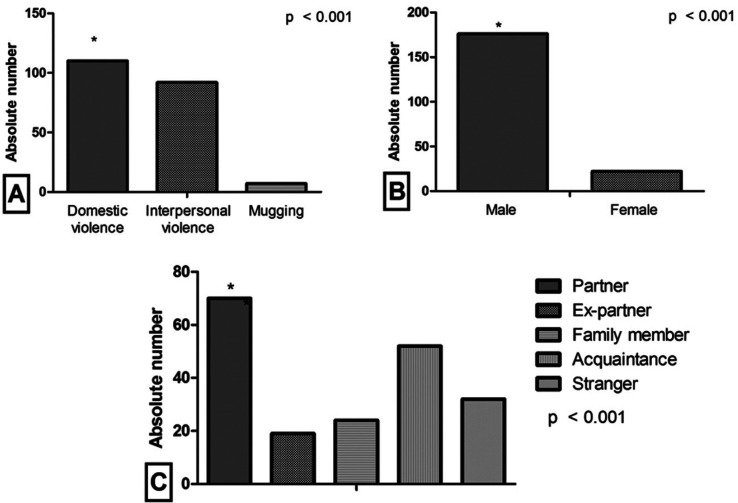
Characterization of the sample according to type of aggression (A) and aggressor (B), and aggressor’s relationship with the victim (C).

Among cases involving aggression (Fig. 1), domestic violence was the most prevalent (n = 110, 52.6%), with male aggressors (n = 176, 88.9%); and of these, most cases were perpetrated by the victim’s partner (n = 70, 35, 5%), and a blunt instrument was the most commonly used weapon (n = 205, 40.8%). These acts of aggression occurred mostly on weekdays (n = 68, 62.4%) and at the nighttime (n = 32, 43.2%).

**Table 1:**
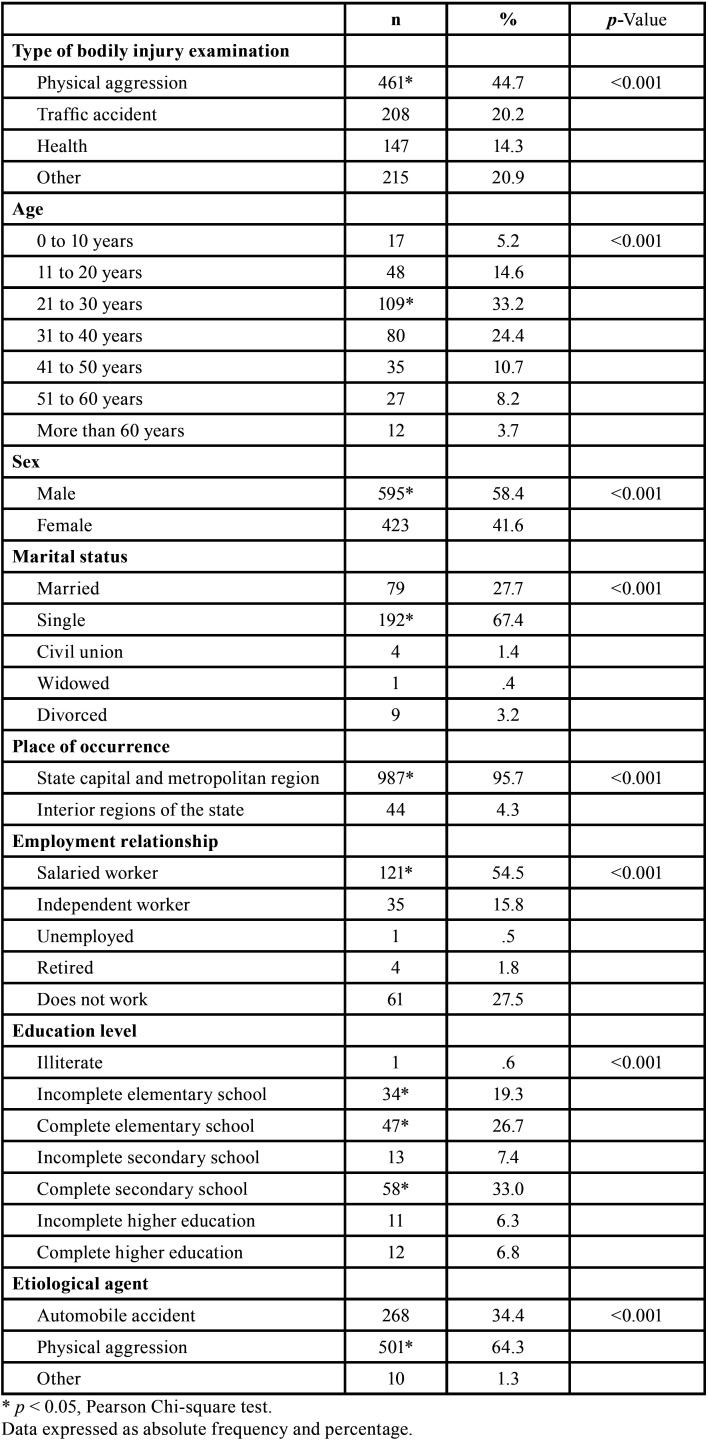
Characterization of the sample according to type of bodily injury examination, age group, sex, marital status, place of occurrence, employment relationship, education level, and etiological agent.

Soft tissue injuries were found in 451 (44.0%) patients, bone fractures in 174 (17.0%) patients, and lesions in the dentoalveolar process were found in 661 (64.6%) patients. Injuries of no forensic-dentistry interest were found in only 76 patients (7.4%). Of the soft tissue injuries, blunt instrument injuries (n = 410, 94.9%) primarily causing blunt-force trauma (n = 174, 40.7%) and bruising (n = 147, 34.4%), and in the upper lip (n = 180, 42.2%) an lower lip (n = 177, 41.5%) (Table 2).

**Table 2:**
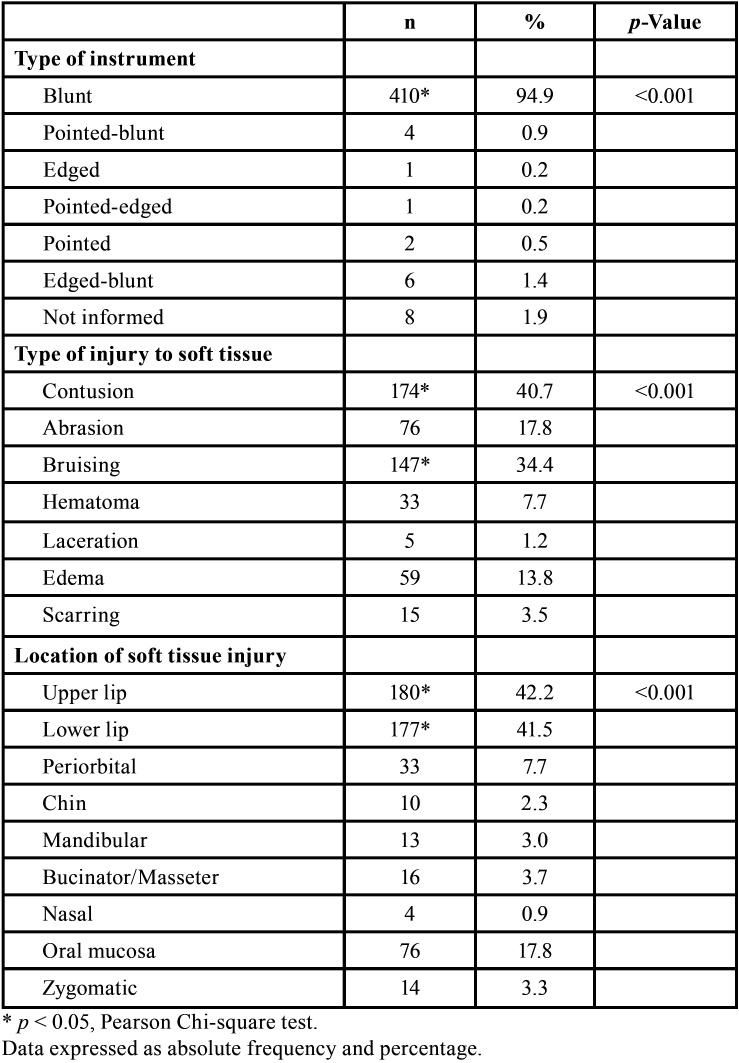
Characterization of the injuries that affected the soft tissue of the face.

Of the injuries with facial fractures, the most prevalent location was the mandible (n = 92, 8.9%), followed by the orbital zygomatic complex (n = 49, 4.8%), and maxilla (n = 43, 4.2%). Of the dental injuries, crown fractures (n = 339, 33.1%), followed by avulsion (n = 149, 14.6%) and mobility (n = 147, 14.4%) were the most commonly observed. Most patients had no traumatized teeth (n = 383, 37.1%); however, 287 (27.8%) and 179 (27.8%), respectively, had one and two traumatized teeth. The maximum number of traumatized teeth was 10 (n = 4, 0.4%) (Table 3).

**Table 3:**
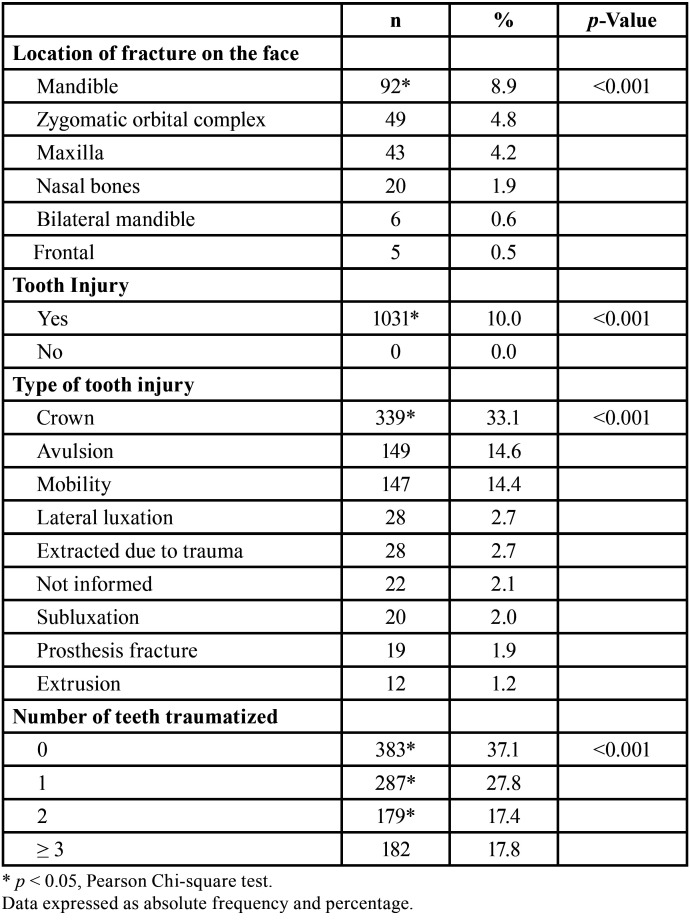
Profile of injuries according to affected bone of the face, presence of traumatized teeth, type of tooth injury, and number of traumatized teeth.

Regarding the forensic ‘experts’ answers to the questions regarding the bodily injury examinations, the item on the existence of an offense to corporal integrity was significantly prevalent (n = 731, 99.5%); regarding the instrument/medium that produced the offense, the most prevalent was a blunt instrument (n = 709, 80.2%). With respect to the item on evaluation of cruelty involved, the most prevalent response was that the injury had not been produced by poison, fire, explosive or torture, or other insidious or cruel means (n = 662, 90.6%). There was a prevalence of responses that the injury did not result in incapacity for usual occupations for more than thirty days (n = 532, 72.9%), and that risk of life was absent in majority of the victims evaluated (n = 714, 92.7%). The items that assessed debility (n = 338, 45.7%) and incapacity/deformity (n = 335, 45.6%) showed a greater need to return for a new examination with a medical report and/or additional examination. When the patient returned for the additional examination, the forensic experts’ responses showed a prevalence that the injuries resulted in incapacity for habitual occupations for more than thirty days (n = 89, 64.5%), with permanent debility, loss or disuse of a limb, sense or function (n = 87, 59.2%), most often resulting in permanent deformity (n = 72, 51.1%).

Risk factors for soft tissue injuries, bone fractures, tooth fractures and alveolar process fractures

Aggression-type bodily injuries were directly associated with soft tissue injuries (*p* < 0.001), while bone fractures showed a direct association with injuries due to traffic accidents and in the additional examinations, and inversely associated with the other types of injuries (*p* < 0.001). Dentoalveolar fractures were not significantly associated with the different types of injuries (*p* = 0.092). Age showed no significant association with soft tissue injuries (*p* = 0.476) or dentoalveolar fractures (*p* = 0.234). However, patients aged 31 to 40 years had a higher frequency of bone fractures. Males were directly associated with bone fractures (*p* < 0.001), and females with soft tissue injuries.

There was no significant difference between gender distribution and prevalence of dentoalveolar fractures (*p* = 0.349). Unmarried individuals showed a higher prevalence of soft tissue injuries (*p* = 0.041), with no difference in bone fractures (*p* = 0.559) or dentoalveolar process fractures (*p* = 0.152). The place of the occurrence and education level did not influence the types of injuries that occurred. Salaried workers showed a higher frequency of bone fractures than the other types of injury (*p* = 0.016).

Automobile accidents were the etiological agent directly associated with bone fractures (*p* <0.001) and inversely associated with soft tissue injuries (*p* < 0.001), the latter having showed a direct association with physical aggression. When an accident was the etiological factor, motorcycle accidents showed a significant association with bone fractures (*p* = 0.001).

The day of the accident, time of the accident and sex of the aggressor showed no significant association with any type of injury. However, when there was aggression, domestic violence was directly associated with soft tissue injuries (*p* = 0.046), and interpersonal violence was directly associated with bone fractures (*p* = 0.001). The aggressor was significantly more often the victim’s partner when the injury was in soft tissue (*p* = 0.022), and an acquaintance when there was a fracture of bone tissue (*p* = 0.040). Firearms were significantly more prevalent in bone fractures (*p* < 0.001) and blunt instruments were more prevalent in soft tissue injuries (*p* = 0.012). The day and time of the aggression did not significantly modify the profile of the injuries.

The mean number of teeth affected was significantly lower when the etiological factor of the physical injury examination was physical aggression (*p* = 0.001). There was no association between number of teeth affected and age (*p* = 0.375), but females presented fewer affected teeth than males (*p* = 0.029). Marital status (*p* = 0.993), locale of occurrence (*p* = 0.168), employment status (*p* = 0.144), and educational level (*p* = 0.189) did not alter the number of affected teeth, but physical aggressions showed a lower mean number of affected teeth than traffic accidents and other types of etiological agent (*p* = 0.001).

The type of accident (*p* = 0.359), day of the accident (*p* = 0.920), time of the accident (*p* = 0.586), type of aggression (*p* = 0.339), sex of the aggressor (*p* = 0.573), aggressor (*p* = 0.739), weapon used (*p* = 0,324), and time of the aggression did not show a significant difference in the number of affected teeth. However, aggressions that occurred on the weekend showed an increase in the number of teeth affected (*p* = 0.033) compared to assaults that took place on weekdays.

In a multinomial logistic regression model, aggression-type injuries (*p* <0.001) and absence of marriage bond (*p* = 0.021) showed a significant association with soft tissue injury, increasing the prevalence thereof 9.78 and 3.33 times, respectively. Bone fractures, in turn, showed a significant association when the physical injury examination was due to traffic accident (*p* < 0.001) and the type of accident was an automobile accident (*p* <0.001), increasing the prevalence of bone fractures 10.75 and 12.09 times, irrespective of the other variables (Table 4).

**Table 4:**
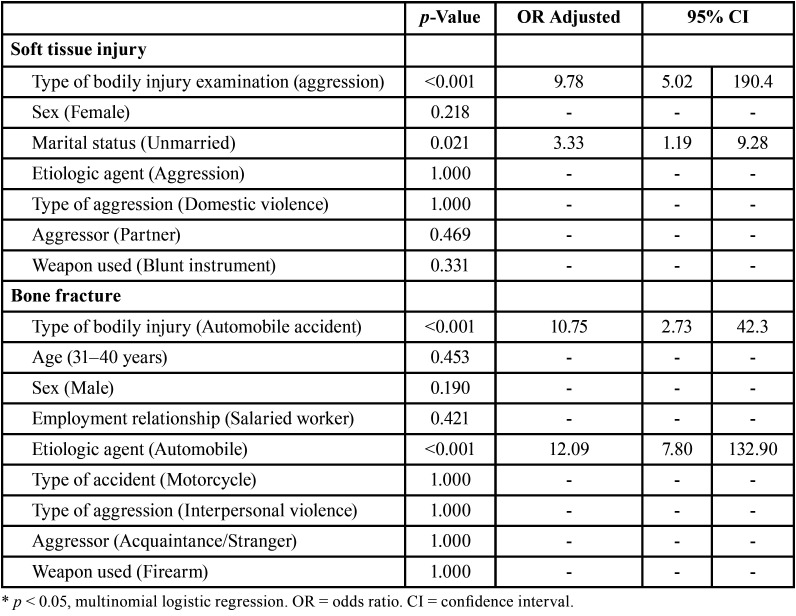
Multivariate analysis for the identification of independent risk factors related to soft tissue injuries and bone fractures of the victims undergoing forensic examination in the present study.

## Discussion

Epidemiological studies on maxillofacial injuries are common with data obtained from major trauma centers around the world, but this type of investigation conducted with records from forensic science centers is considered rare (6). A striking difference between studies from these different sources is the fact that individuals seeking hospital assistance are those who wish to receive medical care, whereas those seeking forensic services expect to obtain evidence for legal proceedings for damages resulting from assault or traffic accident (4,10). This study suggests that cases of facial trauma registered at a forensic unit are not uncommon, and their occurrence is high among young people, especially those in the age range from 21 to 30 years, a fact supported by results from previous studies that show average age between 20 and 29 years (6,11).

In the distribution by sex observed in this investigation, it was verified that the majority of facial injuries occurred in males. Studies have shown that there is a tendency of relation with the etiological agent, whereby males are more commonly affected in cases of traffic accidents and interpersonal violence (12-14). This can be explained by the fact that men participate more actively in social activities and, consequently, they are more susceptible to traffic accidents, work-related accidents, interpersonal violence, and sports-related injuries (13). Women have a higher prevalence of facial injuries when involved with domestic violence (10,12-15), in which their aggressors are most commonly their intimate partners (15-17).

The study by Esses *et al.* (17), which was conducted with data from a university hospital service and carried out by the team of researchers involved in the present study, showed that low educational level, followed by medium educational level, was directly related to the occurrence of maxillofacial fractures. The present study, with a sample obtained from a non-hospital setting, showed a result somewhat similar to the aforementioned study, with a higher incidence of facial trauma for the group of individuals with complete secondary education. This fact reinforces the sociodemographic aspect of maxillomandibular trauma, whether in hospital studies or those developed at forensic centers.

With regard to traffic accidents, the present findings corroborate the current literature, the highest prevalence being those involving motorcycles. The major increase in the number of motorcycles on the streets of Brazilian cities has increased the number of traffic accidents and deaths (8,18). In Brazil, the people undergoing forensic examination, who seek a forensic service due to a traffic accident, request a report with a verification of the existence and quantification of permanent, total or partial injuries in order to file for the compulsory Insurance against Personal Injury Caused by Motor Vehicles (or DPVAT, its acronym in Portuguese). This insurance is a kind of annual contract signed between the Brazilian states and an insurer, and is paid on a mandatory basis by the automobile owners, for the main purpose of indemnifying victims of traffic accidents (9). Several epidemiological studies report that traffic accidents are among the foremost etiological factors of facial trauma (1-9,19,20), and those involving motorcycles are generally the most prevalent (4,5). In contrast, the study by Caldas *et al.* (21) found that accidents involving automobiles were the most prevalent. The prevalence of facial trauma was 41% lower among victims of motorcycle accidents compared to victims of automobile accidents. Although not evaluated in this study, alcohol consumption is an aspect to be considered in the etiology of facial injuries, and may be involved in traffic accidents (1-4).

In cases where physical aggression was the etiological agent, domestic violence was the most prevalent; we can infer that women were the main victims and their companion, the aggressor. This finding is consistent with data reported in other studies that show that when there is a prevalence of violence against women, it is generally practiced by men, either the victim’s husband, boyfriend, or intimate partner (10,16,22,23). The aggressors use physical force, with punches and kicks (the hands and feet are considered blunt instruments) (10,23). In the present study, we observed similar results, in which a blunt instrument was most often used during the assault, with a prevalence of punches. Caldas *et al.* (16) showed that punches, kicks, shoves and slaps were most often used to assault the victims. The use of firearms and other weapons is unusual; however these cases can be interpreted as a clear attempt to kill the victim, considering the potential lethality and destructive power of the used instrument of aggression (13,23).

In this study, most of the violent events occurred during the week, and the most prevalent time was at nighttime. Bernardino *et al.* (14) and Regueira-Diéguez *et al.* (15) presented similar results, in which cases of violence committed by the victim’s intimate partner occurred at nighttime. This finding can be explained by the fact that alcohol and drug consumption is generally higher at night, contributing to an increase in the incidence of interpersonal violence (10,24).

The type of maxillofacial injury observed in the studies has a strong relation with the etiological agent, in which traffic accidents tend to cause more serious injuries, such as bone fractures. In cases associated with physical aggression, there is a pattern of milder injuries, mainly affecting the soft tissues of the face (6,11). In the present study, we observed that traffic accidents were the etiological agent directly associated with bone fractures, and are inversely associated with soft tissue injuries. When a traffic accident was the etiological factor, motorcycle accidents showed a significant association with bone fractures. In the majority of epidemiological studies, mandible fractures are shown to be the primary anatomical site affected in cases of traffic accidents, mainly affecting men (1-9), a result similar to that found in this study.

We have shown that soft tissue was injured by blunt-force traumas causing mainly contusions and bruising most often located on the upper and lower lip, being directly associated to cases of physical aggression, more specifically domestic violence directly affecting unmarried females. In a study conducted in Brazil, based on forensic data, the most prevalent injuries among female victims of domestic violence were edema and hematomas (23). Curca *et al.* (24) observed that hematomas appear more frequently in the periorbital region, followed by the lips and scalp. In this study we observed that of the types of dental injuries, crown fractures, followed by avulsion and mobility were the most commonly observed. Most of the patients had no traumatized teeth. The mean number of affected teeth was significantly lower in the cases of physical aggression, particularly when it occurred on weekdays, and females had fewer affected teeth than males. Caldas *et al.* (21) showed that dental injuries were the second most prevalent type of facial injury, and that tooth loss was the most frequently identified sequela.

## Conclusions

Maxillofacial injuries were significantly associated with sociodemographic and etiological factors in the sample studied. In bodily injury examinations, the majority of persons being examined were young men, salaried workers, primarily exhibiting soft tissue injury and dentoalveolar injury. In the exams related to aggression, the most prevalent type of violence was domestic violence perpetrated by the victim’s partner using a blunt object, directly associated with soft tissue injury. In traffic accidents, the most common type was motorcycle accident, on weekdays, at nighttime, most strongly associated with bone fractures. Thus, this study may contribute to the establishment of preventive public policies for criminal or non-criminal injuries.
